# A Single-Cell Sequencing Guide for Immunologists

**DOI:** 10.3389/fimmu.2018.02425

**Published:** 2018-10-23

**Authors:** Peter See, Josephine Lum, Jinmiao Chen, Florent Ginhoux

**Affiliations:** ^1^Singapore Immunology Network, Agency for Science, Technology and Research, Singapore, Singapore; ^2^Shanghai Institute of Immunology, Shanghai JiaoTong University School of Medicine, Shanghai, China

**Keywords:** single-cell RNA sequencing, MARS-seq, SMART-seq, fluidigm C1, 10X genomics chromium, immunology, dendritic cells

## Abstract

In recent years there has been a rapid increase in the use of single-cell sequencing (scRNA-seq) approaches in the field of immunology. With the wide range of technologies available, it is becoming harder for users to select the best scRNA-seq protocol/platform to address their biological questions of interest. Here, we compared the advantages and limitations of four commonly used scRNA-seq platforms in order to clarify their suitability for different experimental applications. We also address how the datasets generated by different scRNA-seq platforms can be integrated, and how to identify unknown populations of single cells using unbiased bioinformatics methods.

## Introduction

The immune system comprises a network of cells, tissues and organs that mediate host defense against pathogens, but this network also plays a critical role in homeostatic activities, such as tissue development (1), and metabolism (2). With the aid of microscopy and flow cytometry, immune cells can be readily classified into distinct types based on specific surface markers. However, not all immune cell types can be fully resolved by the sole analysis of phenotypic markers, since many of these are expressed by multiple cell lineages, or are differentially regulated during inflammation (3–5). Until recently, gene expression studies were performed on bulk populations of sorted or purified immune cells in attempt to better understand their transcriptomes. During this process, new and unique population markers were identified that can more effectively resolve different immune cell compartments. Nonetheless, this type of analysis does not consider variability in gene expression between individual cells, or the influence of sample contamination with unrelated cell types that share overlapping phenotypic characteristics. Consequently, biologically significant heterogeneity within a population can be masked, and relevant information averaged with irrelevant signals from contaminating cells (6). This is particularly critical when studying temporally dynamic processes, such as progenitor cell development into terminally differentiated populations via multiple transitional stages. Bulk approaches to the analysis of cells that exist in a continuum of differentiation and activation states leads to averaging of their distinct characteristics and a corresponding loss of biologically important information.

Advances in next-generation sequencing technologies have recently made it possible to interrogate the immune system at the level of individual cells. Single-cell RNA-sequencing (scRNA-seq) is now widely employed in immunological studies seeking to resolve previously under-recognized cellular heterogeneity (7, 8), define key processes in cell development and differentiation (9, 10), unravel critical pathways of hematopoiesis (11–13), and understand the gene regulatory networks that predict immune function (14–16). A static snapshot of single-cell transcriptomes can provide a powerful window onto the various stages of differentiation and activation states which are rarely synchronized between cells.

The rapid development of low-input RNA-seq methods has led to an explosion of scRNA-seq protocols, each with their own advantages and limitations. As a result, it is becoming challenging for non-experts to select the most appropriate method to address a specific research question, or to assess whether a single cell approach is even suitable for a given investigation. Here, we list four of the most commonly-used scRNA-seq methods and discuss their strengths and limitations in terms of workflow, sensitivity, data quality, and cost (Table 1), thus providing a guide that could help immunologists make an informed choice for their scRNA-seq studies. We also demonstrated how unbiased single cell identification could be performed, and how data obtained from different scRNA-seq protocols could be integrated prior to downstream analysis.

**Table 1:** Summary of single-cell RNA sequencing methods.

**Method**	**Fluidigm C1 system (SMART-seq)**	**Fluidigm C1 system (mRNA Seq HT)**	**SMART-seq2**	**10X Genomics Chromium system**	**MARS-seq**
cDNA coverage	Full-length	3′ counting	Full-length	5′/3′ counting	3′ counting
UMI	No	No	No	Yes	Yes
Amplification technology	Template switching-based PCR	Template switching-based PCR	Template switching-based PCR	Template switching-based PCR	*in vitro* transcription
Multiplexing of samples	No	Yes	No	Yes	Yes
Single cell isolation	Fluidigm C1 machine	Fluidigm C1 machine	FACS	10X Genomics Chromium single cell controller	FACS
Cell size limitations	Homogenous size of 5–10, 10–17, or 17–25 μM	Homogenous size of 5–10, 10–17, or 17–25 μM	Independent of cell size	Independent of cell size	Independent of cell size
Required cell numbers per run	≥10,000	≥10,000	No limitation	≥20,000	No limitation
Visual quality control check	Microscope examination	Microscope examination	No	No	No
Long term storage	No, must process immediately	No, must process immediately	Yes	No, must process immediately	Yes
Throughput	Limited by number of machines	Limited by number of machines	Limited by operator efficiency	Up to 8 samples per chip	Process is automated
Cost	+ + + + +	+ + +	+ + + +	+	+ +
Sample Preparation Scenario 1 (~5000 single cell)	Targeted cell No: 4992 cells	Targeted cell No: 4800 cells	Targeted cell No: 4992 cells	Targeted cell No: 5000 cells	Targeted cell No: 4992 cells
	26 rounds of 2 runs (2 C1 machines; concurrent)	3 rounds of 2 runs (2 C1 machines; concurrent)	26 rounds of 2 96-well plates	1 run	13 runs of 1 384-well plate
	~26 weeks	~3 weeks	~26 weeks	~2–3 days	~7 weeks
Sample Preparation Scenario 2 (~96 single cell)	Targeted cell No: 96 cells	Targeted cell No: Minimum 800 cell	Targeted cell No: 96 cells	Targeted cell No: Minimum 500 cells	Targeted cell No: 96 cells
	1 run (1 C1 machine)	1 run (1 C1 machine)	1 run of 96-well plates	1 run	1 run of 384-well plate
	~1 week	~1 week	~1 week	~2–3 days	~2–3 days

## Single-cell RNA-sequencing technologies

Since the first scRNA-seq protocol was published in 2009 (17), there has been an expansion of scRNA-seq methods that differ in how the mRNA transcripts are amplified to generate either full-length cDNA or cDNA with a unique molecular identifier (UMI) at either the 5′ or 3′ end. For example, SMART-seq (switching mechanism at 5′ end of RNA template sequencing) (18) and its improved protocol, SMART-seq2 (19, 20) are protocols designed to generate full-length cDNA, while MARS-seq (massively parallel RNA single-cell sequencing) (21), STRT (single-cell tagged reverse transcription) (22, 23), CEL-seq (cell expression by linear amplification and sequencing) (24), CEL-seq2 (25), Drop-seq (26), and inDrops (indexing droplets) (27) are protocols designed to incorporate UMIs into the cDNA. To facilitate automation and ease of sample preparation, some of these protocols can be used together with microfluidic or droplet-based platforms, such as the Fluidigm C1, Chromium from 10X Genomics, and InDrop from 1 CellBio, respectively. The protocols listed here are not comprehensive and alternative scRNA-seq methods have been expertly reviewed in (28–31).

In this review we choose to focus on the following scRNA-seq methods/platforms, namely MARS-seq, SMART-seq2, Fluidigm C1, and 10X Genomics Chromium, as they have been widely used by biomedical scientists in various fields. In addition to their use as standalone technologies, some of these methods can also be combined with fluorescence-activated cell sorting (FACS) which stains cells with fluorophore-conjugated antibodies in order to facilitate separation from a heterogeneous suspension. In particular, it is now possible to “index sort” using FACS to isolate individual cells with known characteristics (e.g., defined size, granularity and selected marker expression), and record their positional location within an assay plate (11). Index sorting allows unexpected questions to be addressed retrospectively since it avoids the use of predefined cell sorting strategies. For example, the phenotype of a rare cell population may not be well-defined, hence an analysis of multiple different markers in various different combinations can help to identify better isolation strategies for downstream experiments. In addition, this approach offers important experimental controls, specifically the ability to determine which cell types are most sensitive to the methodological and technological biases imposed by the protocol e.g., by comparing initial numbers and identities of sorted cells with those that pass later quality controls.

### Massively parallel RNA single cell sequencing (MARS-seq)

MARS-seq is an automated scRNA-seq method in which single cells from the target population are FACS-sorted into 384-well plates that contain lysis buffer (21). The 384-well plates can be stored for long periods prior to sample processing, which allows considerable flexibility with regards to time management. This method is not restricted by cell size, shape, homogeneity or total number. MARS-seq employs a 3′ end-counting mRNA sequencing method which generates partial cDNA transcripts (not full length). The cDNAs are tagged with barcodes together with a unique molecular identifier (UMI) during the initial reverse transcription step, before being pooled and amplified by *in vitro* transcription (IVT). The UMI enables quantitation of the expression levels of individual genes within single cells, thereby reducing the technical variability and bias introduced during the amplification step (23, 32, 33) (which is a distinct advantage over C1 and SMART-seq2 methods, as discussed in more detail later). The pooling strategy enables multiplexing of cDNA amplification, which both simplifies the process and increases sample throughput dramatically. At present, this method is able to detect ~500–3,000 genes per primary cell (Figure S1).

### Fluidigm C1 single cell full length messenger RNA (mRNA) sequencing

The Fluidigm C1 is an automated microfluidic system that can capture and process up to 96 individual cells for relative mRNA quantitation on any Illumina® sequencer. Cell capture, lysis, reverse transcription, and cell multiplexing occur in an integrated fluidic circuit (IFC) chip. Three different cell size cartridges (5–10, 10–17, and 17–25 μM) are available at present, allowing a wide range of cell sizes to be analyzed, although the input cells must be of relatively uniform size and shape in order to avoid selection bias. A minimum of 10,000 cells is required for counts and preparation, making this platform unsuitable for identification of rare populations within a bulk cell sample. The cells to be examined must also be obtained fresh and processed immediately, hence this approach may prove difficult to integrate with experiments that involve long processing times. In addition, since each machine can accommodate only a single cartridge at a given time, multiple machines are required to run multiple cell populations/cartridges concurrently. The high cost of the microfluidic cartridges can also limit the sample size used in each project. Importantly, the C1 system allows captured cells to be individually visualized under the microscope, thereby allowing users to exclude empty wells, doublets, or wells that contain cell debris prior to downstream library preparation. The C1 system employs SMART-sequencing, and generates full-length cDNA (unlike the partial transcripts employed by MARS-seq and 10X Genomics Chromium). C1 technology is currently capable of detecting 300–7,000 genes per primary cell (Figure S1). While the recent introduction of the C1 mRNA Seq HT assay significantly increases system throughput (allowing capture of up to 800 individual cells in a single run), this approach uses 3′ end-counting mRNA sequencing and thus loses read coverage across the entire transcript.

### Switching mechanism at 5′ end of RNA template (SMART-seq2)

SMART-seq2 is the improved version of SMART-seq (similar to Fluidigm C1), featuring refinements to the reverse transcription, template switching, and pre-amplification steps in order to increase yield and length of cDNA libraries generated from each individual cell (while also using off-the-shelf reagents that are available at lower cost) (20). SMART-seq2 generates full-length cDNAs and gives good read coverage across the entire transcript, thereby allowing the detection of gene isoforms or allele-specific expression using single-nucleotide polymorphisms (SNPs). However, UMIs and barcodes cannot be incorporated, hence gene level quantification or multiplexing of samples is not possible, leading to increased complexity of downstream processing. Similar to MARS-seq, individual cells from the target population are sorted into 96- or 384-well PCR plates pre-filled with lysis buffer (hence this method is perfectly compatible with an index sorting approach), and the plates can be stored for long time prior to sample processing. Likewise, SMART-seq2 is not restricted by cell size, shape, homogeneity, or total numbers, making it suitable for experiments that deal with very rare populations. Unlike automated scRNA-seq methods, the reactions are carried out in individual wells which require manual pipetting, thereby making it more time consuming and increasing technical variability. Accordingly, this method may not be the most efficient for experiments that require thousands of individual cells, although liquid handling robots can be used to reduce pipetting issues (albeit at substantially increased cost). Importantly, this method allows a far higher numbers of genes to be detected in each primary cell (~4,000–7,000; Figure S1).

### 10X genomics chromium single cell RNA sequencing

The 10X Genomics Chromium system performs rapid droplet-based encapsulation of single cells using a gel bead in emulsion (GEM) approach. With this method, each gel bead is labeled with oligonucleotides that consist of a unique barcode, a 10 bp UMI, sequencing adapters/primers, and an anchored 30 bp oligo-dT (7). This system allows high throughput and reduces the need for sorting equipment or workflows that involve large numbers of assay plates. Up to eight different samples can be processed simultaneously, making it suitable for experiments that require time course elements or multiple treatments. The downstream processing of individual cells (reverse transcription, cDNA amplification, and library construction) is extremely simple in comparison with the other methods described above, since the reactions for all cells can be performed together in a single tube (rather than requiring the processing of multiple 96-well plates). This platform is currently able to detect 500–1,500 genes per primary cell (Figure S1). While the 10X Genomics Chromium system is the most cost effective and time saving of the methods discussed here, this protocol offers little control over cell input and can be susceptible to selection biases, leading to inaccurate reflection of system biology. Consequently, rare cell populations may not be properly represented if insufficient cell numbers are analyzed. In addition, users are unable to determine which cells are collected prior to downstream processing and quality control measures. This is in contrast to a FACS-based approach where the user knows which cells have been loaded and whether they pass quality control measures. Importantly, the 10X Genomics Chromium system can be used in combination with cellular indexing of transcriptomes and epitopes by sequencing (CITE-seq), a method that allows the detection of multiplexed protein markers with unbiased transcriptome profiling for thousands of single cells (34). Briefly, the cells are stained with antibodies-oligo complexes prior to processing for scRNA-seq. The stained single cells are encapsulated into nanoliter-sized aqueous droplets, lysed in the droplets thereby releasing cellular mRNAs and antibody-derived oligos that anneal via their 3′ poly A tails to gel beads containing oligo-dT, and are indexed by a shared cellular barcode during reverse transcription (34). CITE-seq could be used for studies to study post-translational gene regulation at the single-cell level or even large scale immunophenotyping with large panels of antibodies. Therefore, this may enhance discovery and description of cellular phenotypes, especially cellular populations with subtle transcriptomic differences.

### Considerations for choosing the right platform: biological pragmatism at best!

Advancement in next generation sequencing techniques and computational methods will continue to make scRNA-seq more attractive for general laboratory use. It is clearly paramount to select an appropriate platform for a specific study, but this is highly dependent on the type of biological question being addressed, and is further influenced by the perpetual compromise between cell numbers, information depth, and overall cost (Table 1). A major challenge here is that most investigators will require a reasonable estimate of the level of cellular heterogeneity they expect prior to conducting the experiment.

#### Which protocol should i use?

The choice of which scRNA-seq protocol to use depends on the nature of the research question. Technically, the four approaches described here can be categorized into two groups: full-length methods (SMART-seq2 and Fluidigm C1) and molecular tag-based methods (MARS-seq and 10X Genomics Chromium). Full-length methods cover the entire transcriptome and increase the number of mappable reads, making them suitable for applications including cell-type discovery, assessing tissue composition, allelic gene expression analysis, and even isoform discovery. However, one of the major drawbacks of full-length methods is that they cannot be multiplexed via sample pooling into a single tube for library generation, thereby increasing overall cost and labor. Moreover, UMIs cannot be incorporated to allow digital quantification of the transcripts. In contrast, molecular tag-based methods are based on sequencing of the 5′ or 3′ end of the molecule, hence these can be combined with UMIs to enable multiplexing of samples to improve gene expression quantification and throughput. However, since the reads are restricted to just one end of the transcript, overall sensitivity is reduced compared with “full-length” methods. Despite this drawback, the low cost and high throughput of tag-based approaches means that these are now widely employed in studies of gene expression levels, cell-type discovery, and tissue composition. Platform sensitivity is therefore a critical determinant of sequencing depth and total number of genes detected per cell. The sensitivity of a method is defined as the minimum number of input RNA molecules required for a spike-in control to be confidently detected. Hence, a high sensitivity allows the detection of weakly expressed genes. Two groups have compared the performance in sensitivity, accuracy and cost efficiency of the frequently used scRNA-seq methods (35, 36). Both groups have suggested that 1 million reads per cell is sufficient for saturated gene detection. MARS-seq, Fluidigm C1, and SMART-seq2 was found to detect a median of 4,763, 7,572, and 9,138 genes, respectively (36), which was consistent with what we observed in our analysis of data generated from MARS-seq, SMART-seq2, Fluidigm C1, and 10X Genomics Chromium platform (Figure S1). SMART-seq2 has outperformed the other methods in terms of sensitivity probably due to more mappable reads since the transcripts of tag-based methods may have proximal sequence features that are difficult to align to the genome (30, 36).

#### How many cells do i need to sequence?

Another key consideration of single-cell experimentation is the number of cells required for discovery, which in turn also depends on the specific research objective. For instance, studies that aim to describe the immune landscape or discover rare cell populations can use a breadth-based approach, in which a few hundreds to tens of thousands of cells might be sequenced to provide a reasonable distribution of tissue composition. This type of approach has already been used to map multiple tissues including spleen (21), brain (37, 38), and intestine (39).

One of the pioneering works demonstrated by Amit and colleagues were to dissect the cellular diversity within mouse spleen with the use of MARS-seq (21). From 1536 CD11c^+^ single cells, they identified eight transcriptionally distinct groups that corresponded to B cell, natural killer cell, macrophage, monocyte, and 4 different dendritic cell (DC) subpopulations. In a separate study to map the cellular heterogeneity of the murine brain, 3,005 individual cells from mouse primary somatosensory cortex region S1 and hippocampal region CA1 were sequenced using the Fluidigm C1 platform, in which 47 molecularly distinct subclasses of cells were identified that corresponded to the known major cell types in murine cortex (37). Among these, six different classes of oligodendrocytes were identified, likely representing distinct stages of maturation. Taken together, these studies suggest that the required cell number is dependent on the number of discrete cellular states within the population. In a heterogenous population where the cellular states are transcriptionally distinct and equally distributed, 1,000–2,000 single cells could be sufficient for *de novo* clustering of the different cell states (28). However, if the cell of interest has a distinct transcriptional profile from the mixture of cells, it may be revealed with lesser cells and at a shallower sequencing depth. With the popularity of droplet-based technologies, there will be an increase of low sequencing depth studies that examine 10- to 100-fold more cells (7, 26, 27). Hence, researchers should consider which approach best suits their research questions and budget.

#### What are some potential applications of scRNA-seq?

scRNA-seq has been used in a variety of immunolo gical studies. Traditionally, immune cells have been considered to be homogenous in nature, although some populations may display functional heterogeneity. Recent scRNA-seq studies have revealed that what was once thought to be well-defined immune populations can comprise transcriptionally distinct populations that share overlapping phenotypic markers (9, 40–42). For instance, Bjorklund et al. identified four distinct innate lymphocyte cell (ILC) clusters in human tonsils that corresponded to known phenotypic characterized ILC populations, namely ILC1-3 and natural killer (NK) cells (9). In addition, they also uncovered three transcriptionally and functionally diverse subpopulations within the ILC3 (9). Similarly, Gury-BenAri et al. assessed the heterogeneity of helper-like ILC in the mouse small intestine (40). By combining MARS-seq with chromatin immunoprecipitation-sequencing (CHIP-seq) and assay for transposase-accessible chromatin-sequencing (ATAC-seq), they were able to obtain the transcriptional and regulatory landscape of the cells. Importantly, they revealed that under homeostatic conditions, these helper-like ILC cells showed 15 transcriptional states and a high degree of functional plasticity within the subsets (40). Overall, the studies showed that scRNA-seq can help to reveal cellular heterogeneity that may be masked in traditional phenotypic studies.

scRNA-seq can also be used to profile tissues and aid in the identification of molecular drivers of the disease. This was demonstrated in experimental autoimmune encephalomyelitis in mice. Gaublomme et al. profiled 976 T helper 17 cells with the Fluidigm C1 platform, and showed that these cells were highly heterogeneous and displayed transcriptional signatures that may be correlated with pathogenicity (42). A recent study by Keren-Shaul et al. identified disease-associated microglia (DAM) where they showed DAM interacting and phagocytizing plaques in Alzheimer's disease (41). Such studies can help to better understand the immune responses and pathogenicity of the disease, and pave new roads for the development of new therapeutic agents to treat, manage and even cure the disease.

scRNA-seq can also be used to study immune function, such as antigen receptor repertories. The sequences of T cell receptors can be assembled from scRNA-seq reads and map against a reference pool (43). This was demonstrated by Stubbington et al. who identified various transcriptional states within a single expanded T cell clonotype during *Salmonella* infection in mice (44). A similar tool has also been developed for B cell receptors (45). Such applications will provide a better understanding of how adaptive immunity responds to immune insults, such as infection, autoantigens or vaccination, and spearhead development in therapeutic approaches.

In a developmental context, Giladi et al. recently dissected the differentiation trajectories of hematopoietic stem cells in murine bone marrow, tracking their development into each hematopoietic lineage at single cell resolution (46). This study used MARS-seq to profile gene expression in more than 60,385 individual cells, thus enabling the authors to generate an unbiased reference model of hematopoiesis in normal murine bone marrow. Recognizing the potential of these approaches, the global scientific community has now embarked on an international collaboration using scRNA-seq technologies to establish a “human cell atlas” which maps every cell type in the human body (47). When complete, this atlas will no doubt advance current understanding of human physiology and significantly impact all fields of biology and medicine.

## Case study: using scRNA-seq to resolve dendritic cell ontogeny

A cell type of interest as a case study for this review is Dendritic Cell (DC) as it is small in numbers and heterogeneous in subsets (48). Human peripheral blood mononuclear cells consist of approximately 90% lymphocytes, 10% monocytes, and 1% dendritic cells. In a recent report, scRNA-seq using the 10X Genomics Chromium system was performed on 68,000 unsorted peripheral blood mononuclear cells (PBMC) in order to identify various immune cell populations (7). While this study was able to identify all the major immune cell populations present in blood, the authors found it difficult to identify or resolve cell types whose frequency was less than 1%. Although this type of approach can provide a useful snapshot of the cellular composition of a given tissue, it may be necessary to enrich rare cell types in the sample prior to scRNA-seq, for example by pre-sorting using known or novel surface markers. Indeed, this strategy was recently used by two separate groups to identify human precursors of dendritic cells (pre-DC) in human peripheral blood (8, 10). Villani and colleagues focused on lineage^−^HLA-DR^+^ cells, which comprise known blood DCs and monocytes (8). In their study, the authors performed SMART-seq2 on 2,400 lineage^−^HLA-DR^+^ single cells and detected transcriptionally distinct cell clusters that could be identified using novel surface markers, thus facilitating their isolation by FACS and subsequent analysis by scRNA-seq to validate transcriptional identity. With this method, the authors were able to identify several new types of DCs and monocytes as well as a novel DC precursor population. Separately, our group focused on human blood lineage^−^HLA-DR^+^CD135^+^ cells which consist of both DC subsets and their precursors (10). We performed MARS-seq on 710 lineage^−^HLA-DR^+^CD135^+^ single cells and identified two transcriptionally distinct clusters of plasmacytoid DC (pDC), two subpopulations of conventional DC (cDC), and a new cluster that was later found to constitute pre-DC. Further interrogation of this novel pre-DC population in human bone marrow and peripheral blood revealed that the pre-DC compartment contained distinct lineage-committed sub-populations (one early “uncommitted” CD123^high^ pre-DC subset, and two CD45RA^+^CD123^low^ lineage-committed subsets with distinct functional features). Together, these studies demonstrate that different scRNA-seq platforms can be successfully applied to similar biological questions in complementary ways.

### A computational approach for cell type identification of unknown single cells

Before the emergence of scRNA-seq techniques, cell types were typically defined using a panel of antibodies directed against pre-selected cell surface markers (often guided by prior knowledge of the cell lineage in question and general availability of the relevant antibodies). As technologies have continued to advance, the number of markers per cell that can be measured using flow cytometry or mass cytometry has increased from <10 to >40. This high number of markers allows dissection of cellular heterogeneity in far greater detail, but still lags far behind the level of resolution possible with unbiased methods of cell type identification that employ transcriptomic or proteomic techniques. Indeed, scRNA-seq technologies are now able to measure the transcriptomes of several thousand individual cells in only a short time, and rapid progress in computational methods has made it possible to perform robust identification of these cells in a completely unbiased way. However, a major challenge for biologists after obtaining their scRNA-seq data is knowing how to cluster the data and/or perform cell identification. Many different algorithms are now being used to cluster single cell data, including shared nearest neighbor (SNN) (49), SNN-Cliq (50), pcaReduce (51), clustering through imputation and dimensionality reduction (CIDR) (52), single-cell consensus clustering (SC3) (53), single cell RNA-seq profiling analysis (SINCERA) (54), rare cell type identification (RaceID) (39), GiniClust (55), and single-cell latent variable model (scLVM) (56). After identification of cell clusters, genes that are differentially expressed in each cluster are determined and then assigned as known/novel cell types (based on potentially biased prior knowledge of defining lineage markers).

**Figure 1:**
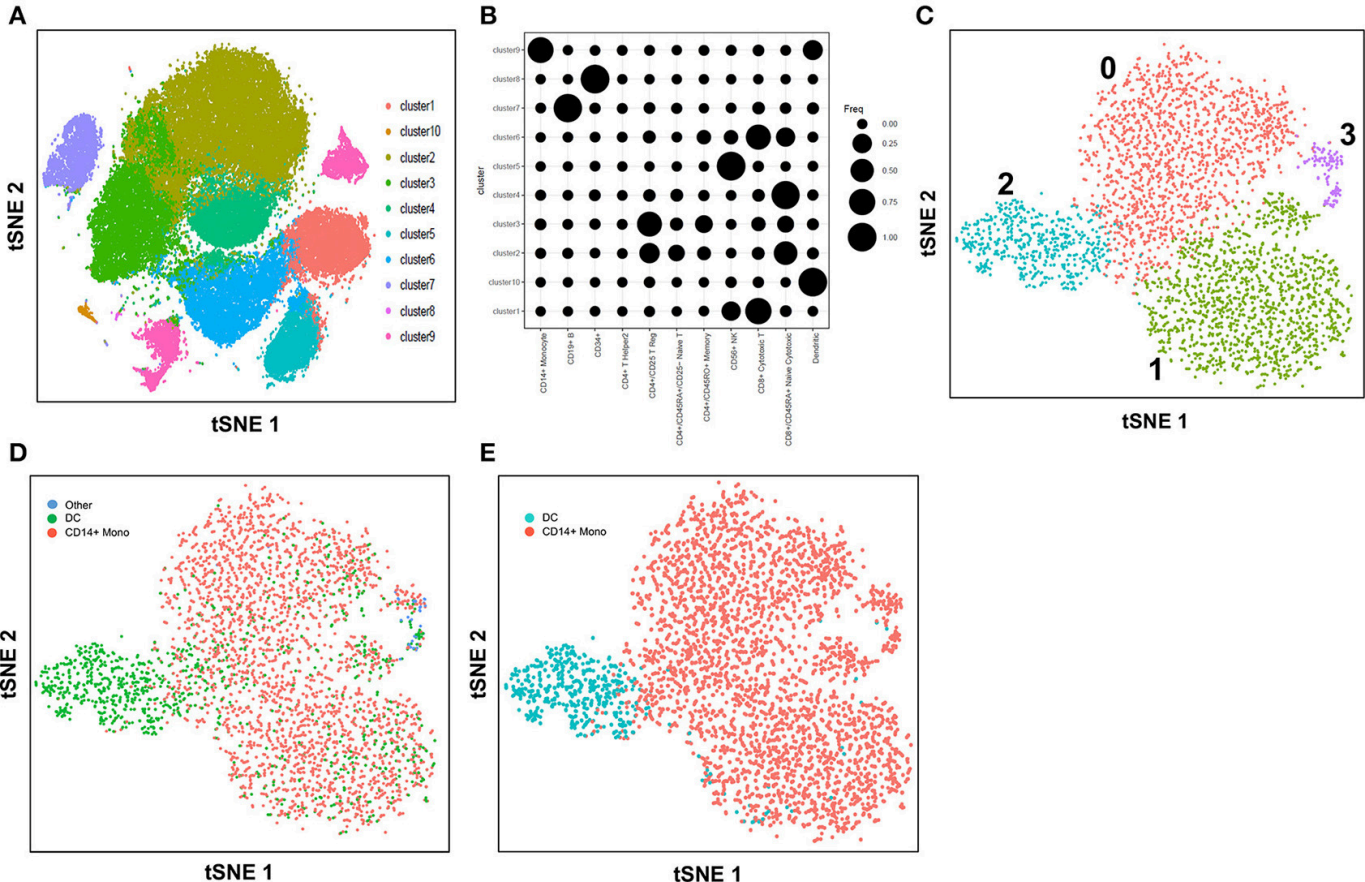
Identification of cell types using scRNA-seq data from 10X Genomics Chromium system. **(A)** tSNE clustering of single cells in PBMC. **(B)** Alignment of clusters to known immune cell populations. **(C)** tSNE clustering of combined cluster 9 and 10 which was inferred as monocytes and DC. **(D)** Superimposed correlation-inferred cell type on the tSNE representation of combined cluster 9 and 10. **(E)** Superimposed CIBERSORT-based cell type classification on the tSNE representation of combined cluster 9 and 10.

Here, we explore the use of the method, cell-type identification by estimating relative subsets of RNA transcripts (CIBERSORT) (57), in an unbiased cell type identification of single-cell transcriptomes, where we analyzed human peripheral blood mononuclear cells (PBMC) scRNA-seq data from two different studies that utilized either the 10X Genomics Chromium (7) or SMART-seq2 (8) platforms. Zheng et al. performed single-cell RNA-seq of 68,000 human PBMC using the 10X Genomics Chromium system (7), then computationally clustered the cells into 10 discrete subsets (Figure 1A), and identified cluster-specific patterns of gene expression. The identity of cell types in each cluster was inferred by aligning cluster-specific genes to known markers of distinct PBMC populations (Figure 1B), as well as by comparing against the scRNA-seq profile of 11 purified PBMC subsets. Single-cell transcriptomes were compared with the average transcriptomes of the 11 purified populations by Spearman's correlation. Each single cell was then assigned the same identity as the purified population with which it had the highest correlation; an approach found to be largely consistent with conventional marker-based methods. For both analyses, cluster 9 was found to contain monocytes and DC, whereas cluster 10 contained DC only. Cells from cluster 9 and 10 were subsequently extracted for further analysis. Cells assigned to cluster 9 segregated into 4 discrete sub-populations when further analyzed using the Seurat package (58) (Figure 1C). These 4 sub-clusters were visually verifiable on the t-Distributed Stochastic Neighbor Embedding (tSNE) reduced dimensions plots. tSNE, as used by Becher et al. to define murine myeloid sub-populations (4), visualizes high-dimensional similarities of cells in a two-dimensional map, which plots cells with similar properties close together, thereby allowing interpretation of each cell type on the basis of location (59, 60). Single cells were initially identified as different lineages via correlation with the purified PBMC populations superimposed on the tSNE plot (Figure 1D). Cluster 2 was found to comprise mainly DC, while clusters 0, 1 and 3 comprised mainly CD14^+^ monocytes. Although correlation-based cell type classification is largely consistent with clustering methods, a number of cells located in monocyte clusters were in fact identified as DC. To resolve whether these cells were indeed monocytes or rather true DC, we performed cell type identification via CIBERSORT analysis (57) using the monocyte and DC gene signatures defined by the single-cell transcriptomic data (i.e., from the 11 purified PBMC subsets). First, we extracted CD14^+^ monocytes and DC and calculated the average gene expression level per cell type. Genes with maximum expression >0.0001 UMI were selected for CIBERSORT analysis, and percentage enrichment of signature genes was calculated for each individual cell, thus allowing assignment of lineage identity according to the most highly enriched gene sets. When this CIBERSORT-based cell type classification was overlaid on the tSNE plot, we observed much higher concordance with both clustering and tSNE segregation (Figure 1E).

**Figure 2:**
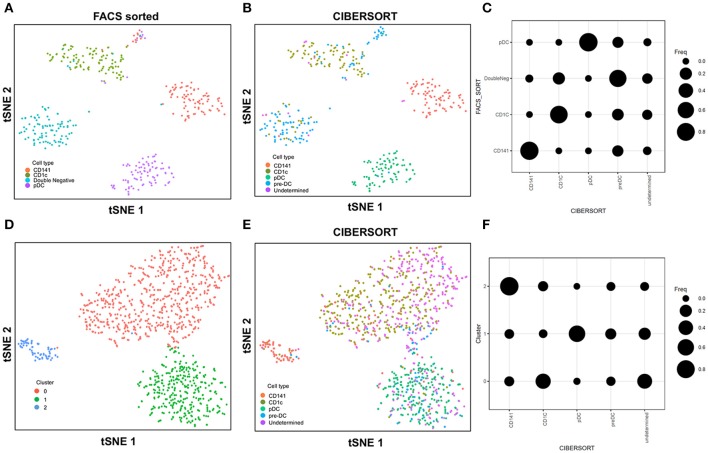
Identification of cell types using scRNA-seq data from SMART-seq2. **(A)** tSNE clustering of dendritic cell subsets. **(B)** Superimposed CIBERSORT-based cell type classification on the tSNE representation of SMART-seq2 dataset. **(C)** Alignment of SMART-seq2 clusters with microarray dataset of DC subsets. **(D)** tSNE clustering of DC cluster derived from 10X Genomics Chromium dataset. **(E)** Superimposed CIBERSORT-based cell type classification on the tSNE representation of DC cluster derived from 10X Genomics Chromium dataset. **(F)** Alignment of DC clusters with microarray dataset of DC subsets.

In this study with the 10X Genomics Chromium system, the use of reference populations of purified PBMC allowed classification of unsorted single cell transcriptomes into 11 major immune cell types. We combined DC from cluster 9 and 10 and then further grouped these into distinct subsets. DCs in human blood are known to comprise two populations of cDC (CD141^+^ cDC1 and CD1c^+^ cDC2) as well as a subset of pDC (5), and a distinct population of pre-DCs (10). In our study (10), we sorted pure populations of CD141^+^ cDC1, CD1c^+^ cDC2, pDCs, and pre-DCs from human blood and generated bulk microarray data, from which we derived characteristic gene signatures for each subset. We next validated these gene signatures against published SMART-seq2 single cell data for each of the four DC populations described in Villani et al. (8). Single cells pooled from each population were subjected to tSNE dimension reduction and then clustered into four subsets using the Seurat package (Figures 2A, S2A). CIBERSORT comparison of these data against microarray-derived gene signatures allowed computational inference of cellular identities that were highly concordant with classification by FACS (Figures 2B,C). Sorted populations of CD141^+^ cDC1, CD1c^+^ cDC2, and pDC were largely assigned to the corresponding cell type by CIBERSORT, with only a small portion of each being classified as pre-DC (likely representing progenitor cells committed to cDC1 or cDC2 fates, as well as uncommitted pre-DC that share phenotypic similarities with pDC). More intriguingly, the majority of sorted double negative cells were predicted to be pre-DCs, suggesting that this compartment may contain genuine cDC precursors. It was not possible to identify some cell types where permutation *p*-values were >0.05. However, despite the fact that cell sorting for microarray and SMART-seq2 was performed by two independent labs, this work confirmed that the signatures derived from microarray were able to aid lineage identification of SMART-seq2 single cells via CIBERSORT. We therefore proceeded to apply the same gene signatures to the prediction of cell types for single cells analyzed with the 10X Genomics Chromium dataset. We first performed tSNE dimension reduction and clustering of individual DC using Seurat (Figure 2D), and overlaid CIBERSORT-inferred cell types on the tSNE plot (Figures 2E,F). Among the 3 clusters generated, cluster 2 comprised predominantly of CD141^+^ cDC1. Unsupervised clustering was in line with cell type inference using sorted cells, suggesting that the conventional marker-based identification of cDC1 is well-defined and can be validated using a marker-free approach. In contrast, cluster 0 represented a mixed population of CD1c^+^ and undetermined cells, whereas cluster 1 comprised a mixture of pDC, pre-DC and undetermined cells. These findings are consistent with earlier reports that CD1c^+^ cDC2 in fact represent a heterogeneous population of poorly characterized composition (8, 10, 61), whereas pDCs are phenotypically similar to pre-DC (hence some progenitor cell functions have likely been mistakenly attributed to pDC because of contaminating pre-DC) (10). Cluster 0 and cluster 1 were assigned as CD1c^+^ cDC2, and pDC, respectively (Figure S2B). Compared with SMART-seq2, the 10X Genomics Chromium dataset generated a higher number of unsorted cells that were labeled as undetermined. These cells were not significantly enriched in signatures of cDC1, cDC2, pDC, or pre-DC, suggesting that these could be unknown subsets acquired by marker-free scRNA-seq of unsorted cells.

In summary, we used two different methods, Spearman's correlation and CIBERSORT, to identify cell types in the 10X Genomics Chromium PBMC dataset. We found that CIBERSORT performed slightly better than did a correlation-based approach. A major reason for this could be that CIBERSORT first identified signature genes for each cell type, followed by an additional step of vector regression to calculate a gene signature enrichment score. In any case, both methods use bulk transcriptomes for reference and are thus highly dependent on the cell types present in the reference dataset. Accordingly, the use of a comprehensive dataset that is directly relevant to the study of interest will significantly improve the accuracy of cell type identification.

### Data integration and correction of technical variation

**Figure 3:**
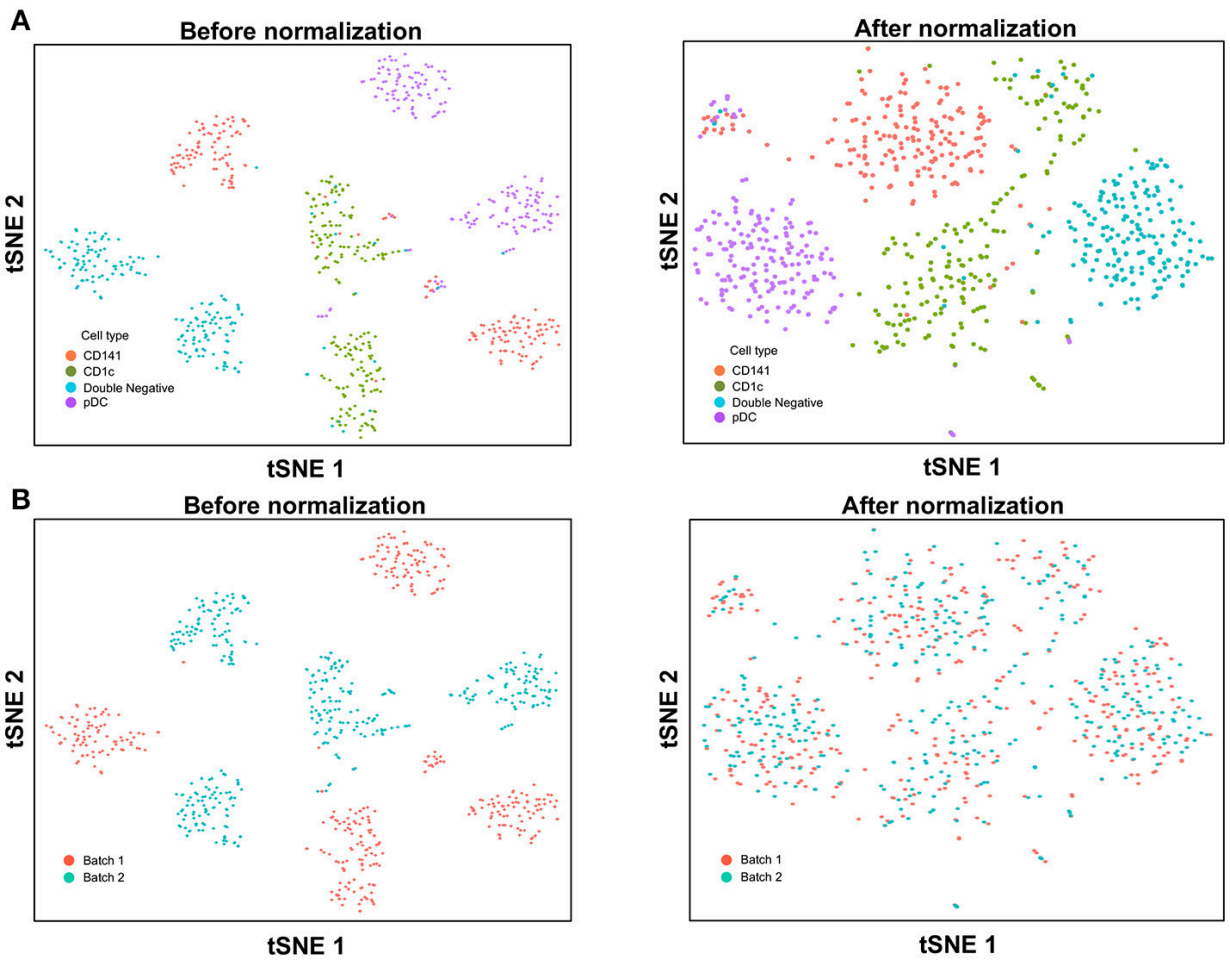
Batch effect correction of SMART-seq2 dataset. **(A)** Batch effect was observed in two separate SMART-seq2 datasets before CCA normalization, but this was absent after application of CCA normalization. **(B)** Cell clusters corresponded to the batch of SMART-seq2 dataset before CCA normalization. After CCA normalization was applied, both batches of single cells overlapped with each other.

With the increased data yield provided by scRNA-seq, researchers can now mine existing datasets to perform multiple different types of analysis. However, the datasets generated by different scRNA-seq platforms often require integration prior to downstream analysis, and technical variation between datasets must be corrected before these can be combined. When applying scRNA-seq to a large number of cells, the experiments are usually carried out in batches, resulting in prominent inter-assay variability that can conceal biological heterogeneity. For example, Villani et al. performed SMART-seq2 on two separate batches of sorted cDC1, cDC2, double negative DC, and pDC (8), before performing t-SNE dimension reduction and clustering analysis, which identified two distinct sub-populations for each input cell type (Figure 3A). Overlaying batch information onto the tSNE plot revealed that these sub-populations corresponded to the two separate assay runs (Figure 3B). To remove this batch effect, the Seurat package implements the canonical correlation analysis (CCA) algorithm, which identifies the dimensions in which batch 1 and 2 have the highest correlation and projects the cells onto these dimensions. After CCA normalization, the same cell types from batch 1 and batch 2 were well-aligned (Figure 3A), with no evident separation of cells between assay runs (Figure 3B).

**Figure 4:**
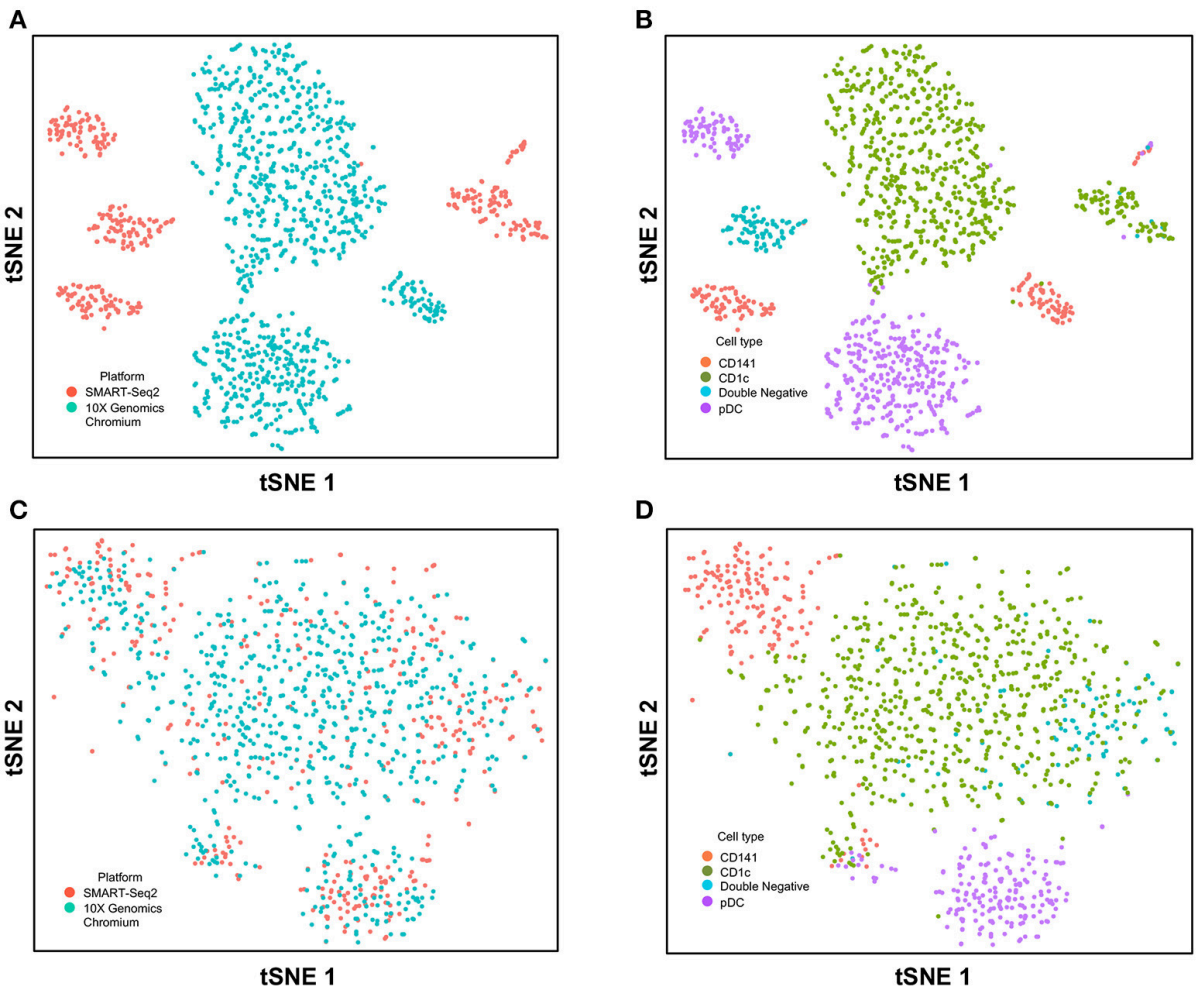
Correction of technical variation in DC subset dataset from 10X Genomics Chromium and SMART-seq2 datasets. **(A)** tSNE clustering of SMART-seq2 and 10X Genomics Chromium dataset. **(B)** Cell type identification in the combined tSNE clusters of SMART-seq2 and 10X Genomics Chromium dataset. **(C)** CCA normalization of DC subsets from SMART-seq2 and 10X Genomics Chromium dataset. **(D)** Identification of cell types after CCA normalization.

**Figure 5:**
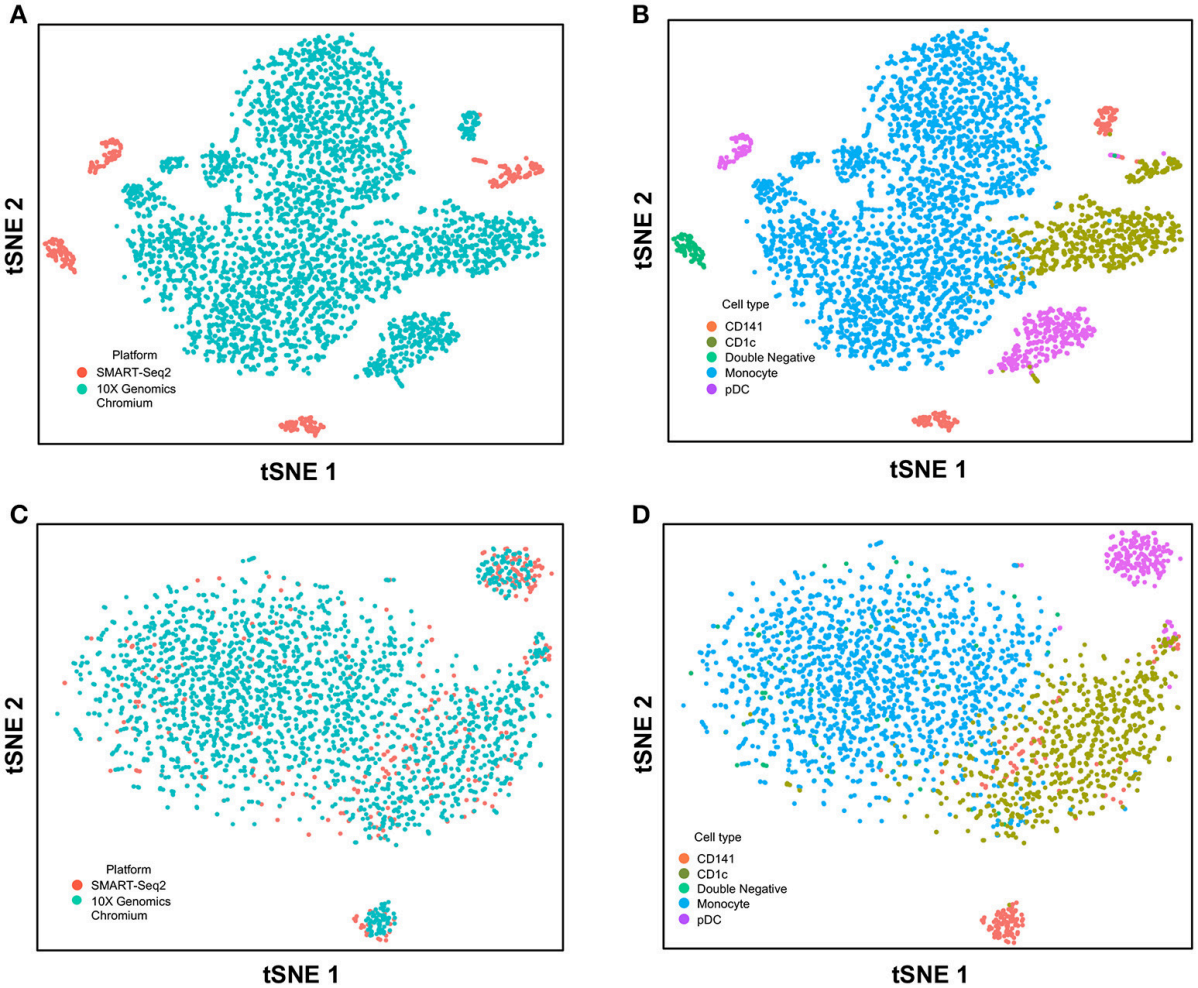
Correction of technical variation in monocytes and DC subset dataset from 10X Genomics Chromium and SMART-seq2 datasets. **(A)** tSNE clustering of SMART-seq2 and 10X Genomics Chromium datasets. **(B)** Cell type identification in the combined tSNE clusters of SMART-seq2 and 10X Genomics Chromium datasets. **(C)** CCA normalization of monocytes and DC subsets from SMART-seq2 and 10X Genomics Chromium datasets. **(D)** Identification of cell types after CCA normalization.

Next, we used CCA to integrate single-cell data as generated by SMART-seq2 method and 10X Genomics Chromium system. Single cells isolated from purified cDC1, cDC2, double negative cells, and pDC populations were prepared using SMART-seq2. Single cell data from unsorted PBMCs were generated by 10X Genomics Chromium system and only DC were isolated for integration with SMART-seq2 data. DCs from the 10X Genomics Chromium experiment were inferred based on CIBERSORT analysis as mentioned previously. Before CCA normalization, cells from SMART-seq2 method and 10X Genomics Chromium system were well-separated (Figure 4A), and two distinct subsets were identified for each lineage, reflecting the use of the two different analytical platforms (Figure 4B). After CCA normalization, the cells analyzed by each platform became well-mixed (Figure 4C) and were clustered mainly by cell type (Figure 4D). Notably, the double negative cells that were previously separated from other lineages were also observed to merge with the CD1c^+^ population after CCA. We next attempted to integrate datasets of slightly different cellular composition by adding monocytes to the 10X Genomics Chromium data, whereas the SMART-seq2 dataset still comprised DC only. Cells were clustered mainly by cell type regardless of the platform used, except that double-negative DCs were now allocated to the monocyte cluster and some CD141^+^ cells were now present in the CD1c^+^ cluster (Figure 5).

Our analysis indicates that CCA is able to correct batch effect confounders when no other biological factors differ between experimental replicates. The CCA algorithm make the assumption that data from both batches have the same or similar cellular composition. It is important to note that CCA can still force batches to align even if they have dissimilar cellular composition, which can result in masking of genuine biological variation. To overcome this limitation, the mutual nearest neighbor (MNN) algorithm (62) can be employed to identify similar cell populations or “pairs” that are present in both batches. MNN pairs are used to calculate analytical drift between assay runs and subsequently compensate batch effect for all cells present. In the absence of any shared structure, a cell population of known composition (e.g., a cell line) can also be spiked into each sample in order to remove batch effects by providing a uniform reference population. While both CCA and MNN are powerful tools, several other normalization techniques (both current and future) may further improve batch effect correction in the years ahead. However, a thorough comparison of these novel methods will be required using variable input data in order to identify which approaches best suit which datasets.

## Concluding remarks

In this review, we discussed the relative strengths and limitations of some widely-used scRNA-seq platforms, as well as current technical barriers to analyzing single-cell transcriptome datasets. As next generation sequencing techniques and computational methods continue to improve, the use of scRNA-seq in immunological studies will become more widespread and eventually even routine. Once a complete set of reference databases or “immune mapping” studies has been completed, new strategies will be required to multiplex single-cell profiling with other techniques that permit analysis of multiple molecular features of individual cells in parallel (63–65). As the complexity of these technologies increases, investigator choice of analytical platform must be carefully guided by specific hypotheses and biological questions, hopefully leading to deeper insight into the role of the immune system in health and disease.

## Data availability statement

The datasets analyzed for this study can be found in the Gene Expression Omnibus under accession numbers GSE80171 (https://www.ncbi.nlm.nih.gov/geo/query/acc.cgi?acc=GSE80171), GSE94820 (https://www.ncbi.nlm.nih.gov/geo/query/acc.cgi?acc=GSE94820), and 10X Genomics Chromium system single cell gene expression datasets (https://support.10xgenomics.com/single-cell-gene-expression/datasets).

## Author contributions

PS, JL, JC, and FG wrote the manuscript. JC performed data analysis of the scRNA-seq datasets.

### Conflict of interest statement

The authors declare that the research was conducted in the absence of any commercial or financial relationships that could be construed as a potential conflict of interest.
