# Corrigendum: A Single-Cell Sequencing Guide for Immunologists

**DOI:** 10.3389/fimmu.2019.00278

**Published:** 2019-02-26

**Authors:** Peter See, Josephine Lum, Jinmiao Chen, Florent Ginhoux

**Affiliations:** ^1^Singapore Immunology Network, Agency for Science, Technology and Research, Singapore, Singapore; ^2^Shanghai Institute of Immunology, Shanghai JiaoTong University School of Medicine, Shanghai, China

**Keywords:** single-cell RNA sequencing, MARS-seq, SMART-seq, fluidigm C1, 10X genomics chromium, immunology, dendritic cells

In the original article, there was an error. The statement “Human peripheral blood consists of ~90% lymphocytes, 10% monocytes and 1% dendritic cells” is incorrect.

A correction has been made to the ***Case study: Using scRNA-seq to resolve dendritic cell ontogeny***:

“A cell type of interest as a case study for this review is Dendritic Cell (DC) as it is small in numbers and heterogeneous in subsets (48). Human peripheral blood mononuclear cells consist of approximately 90% lymphocytes, 10% monocytes, and 1% dendritic cells. In a recent report, scRNA-seq using the 10X Genomics Chromium system was performed on 68,000 unsorted peripheral blood mononuclear cells (PBMC) in order to identify various immune cell populations (7). While this study was able to identify all the major immune cell populations present in blood, the authors found it difficult to identify or resolve cell types whose frequency was less than 1%. Although this type of approach can provide a useful snapshot of the cellular composition of a given tissue, it may be necessary to enrich rare cell types in the sample prior to scRNA-seq, for example by pre-sorting using known or novel surface markers. Indeed, this strategy was recently used by two separate groups to identify human precursors of dendritic cells (pre-DC) in human peripheral blood (8, 10). Villani and colleagues focused on lineage^−^HLA-DR^+^ cells, which comprise known blood DCs and monocytes (8). In their study, the authors performed SMART-seq2 on 2,400 lineage^−^HLA-DR^+^ single cells and detected transcriptionally distinct cell clusters that could be identified using novel surface markers, thus facilitating their isolation by FACS and subsequent analysis by scRNA-seq to validate transcriptional identity. With this method, the authors were able to identify several new types of DCs and monocytes as well as a novel DC precursor population. Separately, our group focused on human blood lineage^−^HLA-DR^+^CD135^+^ cells which consist of both DC subsets and their precursors (10). We performed MARS-seq on 710 lineage^−^HLA-DR^+^CD135^+^ single cells and identified two transcriptionally distinct clusters of plasmacytoid DC (pDC), two subpopulations of conventional DC (cDC), and a new cluster that was later found to constitute pre-DC. Further interrogation of this novel pre-DC population in human bone marrow and peripheral blood revealed that the pre-DC compartment contained distinct lineage-committed sub-populations (one early “uncommitted” CD123^high^ pre-DC subset, and two CD45RA^+^CD123^low^ lineage-committed subsets with distinct functional features). Together, these studies demonstrate that different scRNA-seq platforms can be successfully applied to similar biological questions in complementary ways.”

The authors apologize for this error and state that this does not change the scientific conclusions of the article in any way. The original article has been updated.

