# Supplementary material for: A Single-Cell Sequencing Guide for Immunologists
Source: Front Immunol. 2018 Oct 23;9:2425. doi: 10.3389/fimmu.2018.02425 (PMC6205970; doi:10.3389/fimmu.2018.02425)
Supplement: Supplementary file 1 [file Table_1.DOCX]

Supplementary Material

A Single-Cell Guide for Immuno-Dummies

Peter See^1^, Josephine Lum^1^, Jinmiao Chen^1,^* and Florent Ginhoux^1,2,^*

*** Correspondence:** Corresponding Author: [florent_ginhoux@immunol.a-star.edu.sg](mailto:florent_ginhoux@immunol.a-star.edu.sg) and chen_jinmiao@immunol.a-star.edu.sg

## Supplementary Figures


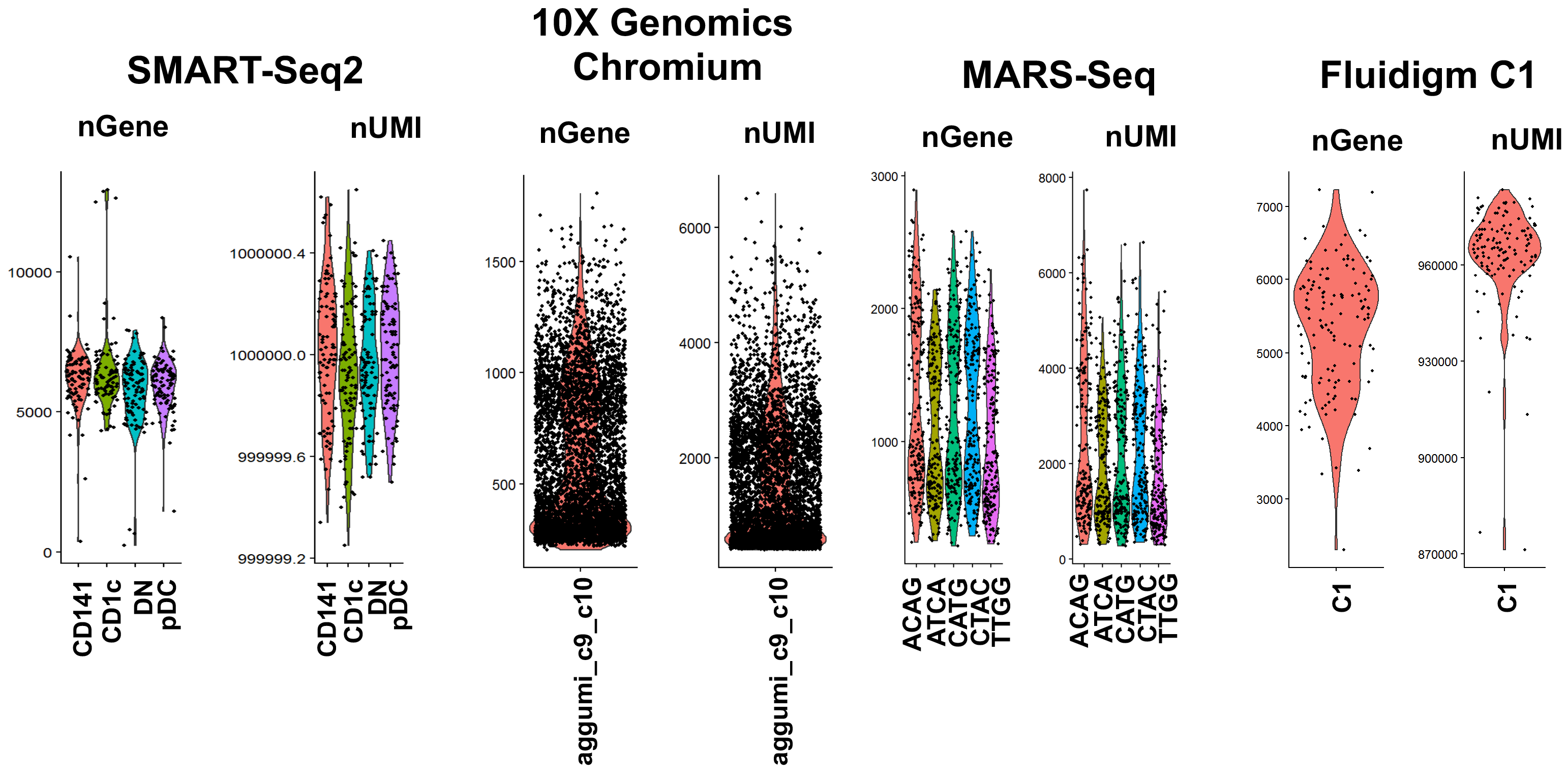


Figure S1: Number of genes and UMI detected in each of the scRNA-seq platforms.


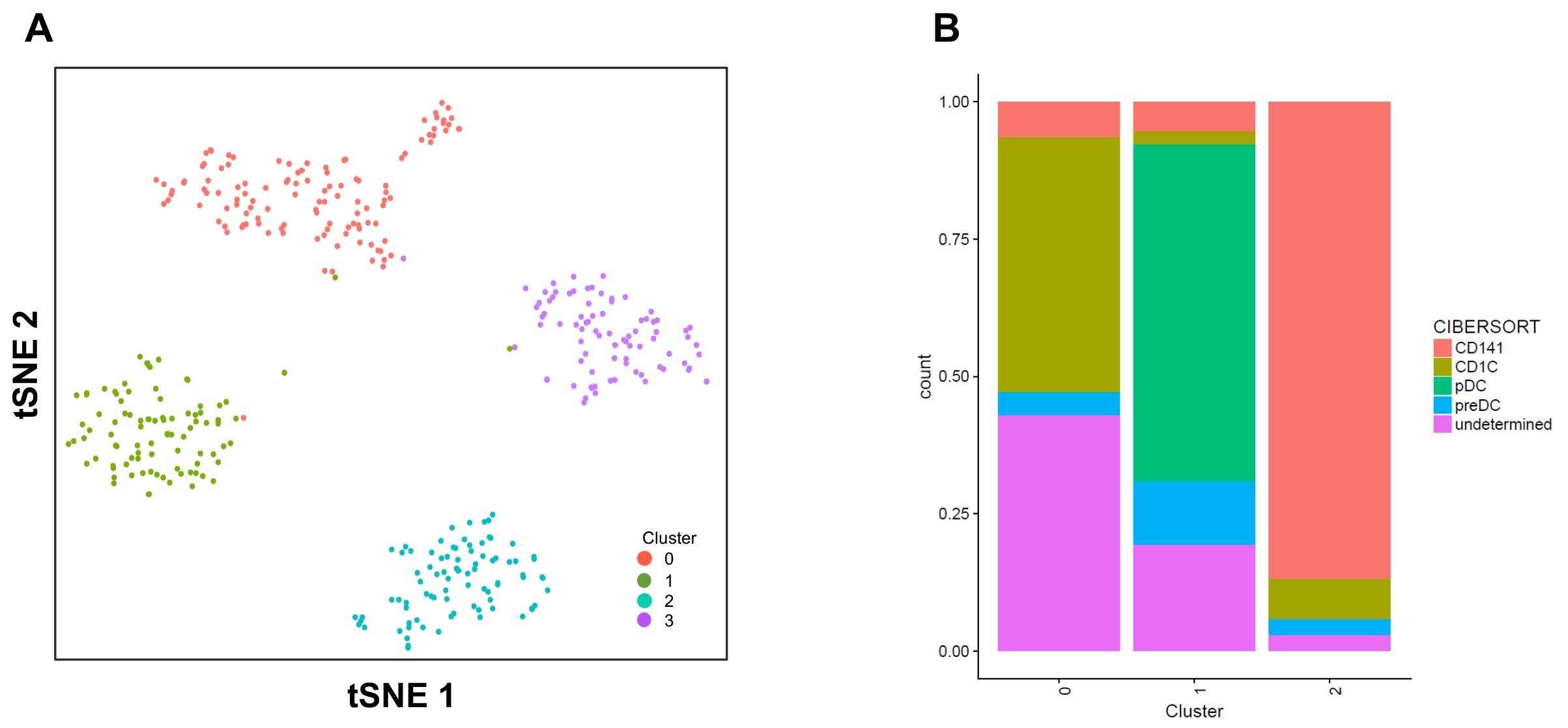


Figure S2: Identification of cell types using scRNA-seq data from SMART-Seq. (A) tSNE clustering of dendritic cell subsets. (B) CIBERSORT analysis determines the frequency of each of the DC subsets in the 3 DC clusters defined from 10X Genomics dataset.
